# Supplementary material for: Zebrafish Slit2 and Slit3 Act Together to Regulate Retinal Axon Crossing at the Midline
Source: J Dev Biol. 2022 Sep 23;10(4):41. doi: 10.3390/jdb10040041 (PMC9590056; doi:10.3390/jdb10040041)

Figure S1

A

***slit3* genomic sequence**

1
ATCAGCCTCAGAGGATATATG**ATG**CGGTGGGTAAAGTATTGGGGGACAGTGGCTGTGCTCTGCGGGCTGCTGAGCTGT

GCTGTGAATGGATGCCCGCATAAGTGCAGTTGTTCGGGCTCTCATGTGGATTGTCAGGGTCAAGCCTTCAAGACGGTG

167 13712
CCTAGGGGAATCCCGAGGAATGCCGAGAGACT//CCCTGTGGGC**ATCTGGTGCTGTTGAGGCAC**TGCCAGGGAGCCTC

TGTGTTTAAGATCTCTGTTTACTGACTCTGCTGTGAGCTTAGACTTGAAAAATCATGTTTGAAAGCATAAGTGAAATG

AAAAGCTTTTAACTTCTCTTAACCATCCTCTCTCCCCTTTCTCTTTCCAGGGATCTGGACAGGAATAACATCACCCGG

13919
ATCACCA**AAGTGGACTTCTCTGG**CCTCAAGAACCTGCGCGTGCTGTAAGATGTCACTCTGTATCTCTCACTCTCATGG

TTAATCCATCCTTTAACTCCCAAGTGCCCCTCCTCTTCCTTGTTGTCTTTATTGTCAAAGCCATCAAAGGGTTCGAAG

CTGAAA**GCTGGGAGGATTACAGCACA**AGTAATAATAAAACAAACTGACATTTCAGCTGAGTAAATCTGACACATTTAA

B

***slit3* mRNA**

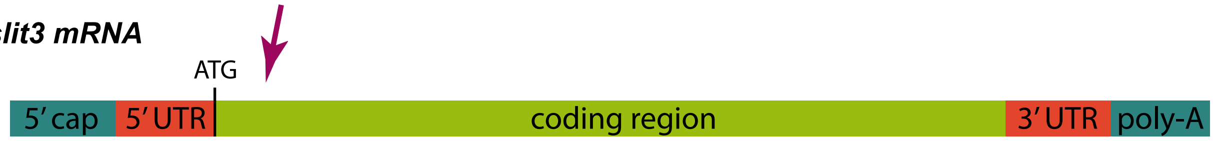

1
ATGCGGTGGGTAAAGTATTGGGGGACAGTGGCTGTGCTCTGCGGGCTGCTGAGCTGTGCTGTGAATGGATGCCCGCAT

AAGTGCAGTTGTTCGGGCTCTCATGTGGATTGTCAGGGTCAAGCCTTCAAGACGGTGCCTAGGGGAATCCCGAGGAAT

206
GCCGAGAGACTGGATCTGGACAGGAATAACATCACCCGGATCACCA**AAGTGGACTTCTCTGG**CCTCAAGAACCTGCGC

C

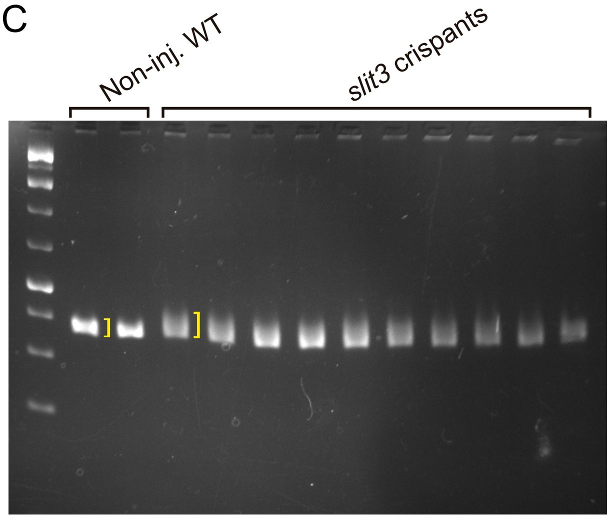

D

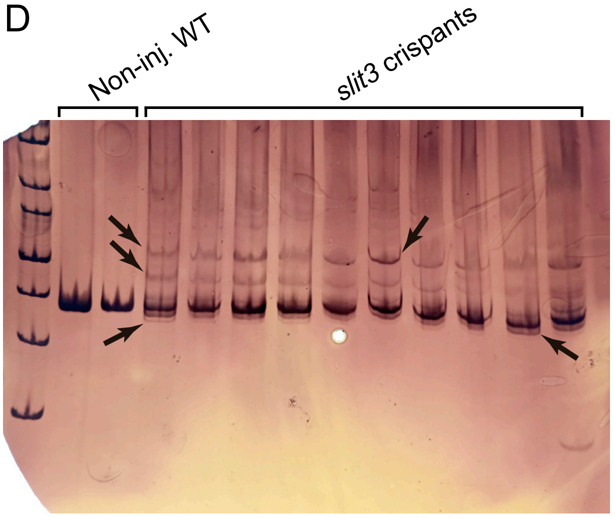

Supplement: Supplementary file 1 [file jdb-10-00041-s001.zip › jdb-1888759-supplementary/Fig. S1rev.pdf]
