# Supplementary material for: Zebrafish Slit2 and Slit3 Act Together to Regulate Retinal Axon Crossing at the Midline
Source: J Dev Biol. 2022 Sep 23;10(4):41. doi: 10.3390/jdb10040041 (PMC9590056; doi:10.3390/jdb10040041)
Supplement: Supplementary file 1 [file jdb-10-00041-s001.zip › jdb-1888759-supplementary/SupplTables-Legends Davison22.pdf]

SUPPLEMENTARY TABLES

Table S1. Single-guide RNA sequences used for CRISPR/Cas9 mutation.

**sgRNA*slit2* 71**	TAATACGACTCACTATAGGGCTCCTGCAGCGGGACAGGTT TTAGAGCTAGAA
sgRNA*slit3* 133	TAATACGACTCACTATAGGGTGCCTAGGGGAATCCCGGTT TTAGAGCTAGAA
**sgRNA*slit3* 206**	TAATACGACTCACTATAGGGGCCAGAGAAGTCCACTTGTT TTAGAGCTAGAA
sgRNA*slit3* 324	TAATACGACTCACTATAGGAGGACCTGAAGCCGGTTCGTT TTAGAGCTAGAA
sgRNA*slit3* 653	TAATACGACTCACTATAGGTTGGCTGCGGCAGAGGAGGTT TTAGAGCTAGAA

Table S2. Primer sequences used for quantitative PCR analyses.

eIF1a qPCR - forward primer	CTACCTACCCTCCTCTTGGTCG
eIF1a qPCR - reverse primer	ACACGCCGCAACCTTTGGAA
slit2 qPCR - forward primer	GCCCCAGTCACACTCATCC
slit2 qPCR - reverse primer	CTGGCAGGTTGGTGGGTATT
slit3 qPCR - forward primer	TTTGGCCCCTTTCACACAGT
slit3 qPCR - reverse primer	TCTACGGGACCTGTGCAAAC
slit3 206 genotyping - forward primer	ATCTGGTGCTGTTGAGGCAC
slit3 206 genotyping - reverse primer	TGTGCTGTAATCCTCCCAGC

SUPPLEMENTARY FIGURE AND VIDEO LEGENDS

Figure S1. Injection of sgRNA 206 together with nCas9n induces indels in the *slit3* gene target site. A. Partial sequence corresponding to the zebrafish *slit3* gene. Exons are represented in black, while introns are coloured in grey. The initiation codon (ATG) is marked in bold. The entirety of the first exon is shown, while only the 3'-most region of the first intron can be observed, with the double bar indicating an unshown sequence belonging to the first intron. The *slit3* 206 sgRNA-binding site is shown in bold and coloured in purple, while the sequences complementary to the primers used for genotyping are underlined. **B.** Schematic representation of the *slit3* mRNA. The arrow indicates the approximate site of expected mutagenesis. The coding sequence of *slit3* is also shown, with the sgRNA-binding site indicated in bold purple. **C,D.** Agarose (**C**) and polyacrylamide (**D**) gel electrophoresis analyses showing PCR products obtained from genomic DNA. The same samples were run in both gels, with each lane corresponding to an individual embryo. Two non-injected wild-types and ten *slit3*crispants were analyzed. Brackets in the agarose gel indicate the difference in band width between wild-type and crispant embryos; arrows in the polyacrylamide gel indicate extra bands, not observed in wild-types.

Figure S2. Analysis of the bifurcation phenotype in *slit2-/-^{mz}* and *slit3*crispant embryos. A,B. Maximum intensity z-projections of the optic chiasm region of 48 hpf embryos immunostained to label RGCs (zn8 antibody; ventral view). Four different phenotypes can be seen in *slit2-/-^{mz}* (**A**) and *slit3* crispant (**B**) embryos: no bifurcation, asymmetrical bifurcation with a thin anterior branch, asymmetrical bifurcation with a thin posterior branch, and symmetrical bifurcation with branches of similar thickness. The number of cases observed for each phenotype is shown. Arrows indicate the site of bifurcation. Scale bar: 30 μm.

Figure S3. Axon turn angles across the optic chiasm. The angle with respect to the horizontal axis (medio-lateral) was determined for each time point, from each axon on the tracking analysis shown in Fig. 5A. All angles obtained for each experimental

condition were plotted together in this graph, to compare general angle distributions and mean ± SD. Positive angles indicate dorsal turn, and negative angles ventral turn. Angle means are not significantly different (Brown-Forsythe and Welch test), and the mean value for the non-injected wild-type embryos is around 0.5°, following the curvature of the ventral diencephalon.

Video S1. Simultaneous loss of Slit2 and Slit3 causes severe axon guidance defects at the optic chiasm. Z-stacks showing the optic chiasm of non-injected wild-type, *slit2-/-^{mz}*, *slit3* crispant and *slit2-/-^{mz};slit3* crispant embryos at 48 hpf, after immunostaining with zn8 antibody. In non-injected wild-type embryos, one optic nerve can be seen crossing anteriorly to the contralateral nerve, while in *slit2-/-^{mz}* and slit3 crispant embryos, one of the optic nerves splits and surrounds the contralateral nerve in ≈25 % of the cases. *slit2-/-^{mz};slit3* crispant embryos display more severe defects, which include projections to the anterior telencephalon. The stack sequence is shown from the ventral to the dorsal region of the embryo. Corresponding to Fig. 1A.

Video S2. Simultaneous loss of Slit2 and Slit3 causes retinal axon misprojections at the optic chiasm. 3D projections of z-stacks of the optic chiasm of non-injected wild-type, *slit2-/-^{mz}*, *slit3* crispant and *slit2-/-^{mz};slit3* crispant embryos observed at 48 hpf, where RGC axons from both eyes were labeled anterogradely with either DiI or DiO. In non-injected wild-type embryos, one of the optic nerves can be seen crossing anteriorly and slightly ventral to the contralateral nerve, while remaining physically separate. In *slit2-/-^{mz}* and in *slit3* crispant embryos, one of the optic nerves can be seen splitting into two groups of axons which surround the contralateral nerve. More severe defects can be seen in *slit2-/-^{mz};slit3* crispant embryos, including projections to the ipsilateral optic tract and the contralateral optic nerve. Corresponding to Fig. 2.

Video S3. RGCs in *slit2/slit3* double crispants partially project to the ipsilateral optic tectum. 3D projection of z-stack of the optic tectum of an *atoh7*:GFP transgenic *slit2+slit3* crispant 5 dpf larva, where RGC axons from one eye were labeled

anterogradely with DiI. The tectum ipsilateral to the labeled eye is shown, where DiI-labeled axons can be seen. Corresponding to Fig. 3D,d’.

Video S4. Retinal axons from *slit2*, *slit3* and *slit2/slit3* double crispants present navigation errors around the midline. Confocal time-lapse (4D) maximum intensity projection images of the optic chiasm region from *atoh7*:GFP transgenic embryos, acquired every 15 min from 30 hpf. A representative non-injected wild-type, *slit2* crispant, *slit3* crispant and *slit2+slit3* crispant are shown. At the end, a full stack reconstruction of the methyl green-counter-stained embryos 16.5 h after the beginning of the time-lapse experiments is shown. Ipsilateral turns and minor pathfinding errors can be seen in *slit2* crispant, *slit3* crispant and *slit2+slit3* crispants, but not non-injected wild-types. At the end of the experiment, these optic chiasms show clear defects in the sorting of axons coming from each eye. Corresponding to Fig. 4; see also Videos S5-8.

Video S5. Retinal axons from *slit2*, *slit3* and *slit2/slit3* double crispants present navigation errors around the midline II. Confocal time-lapse (4D) maximum intensity projection images of the optic chiasm region from an *atoh7*:GFP transgenic embryo injected with *slit2* sgRNA/nCas9n, acquired every 15 min from 30 hpf. At the end, a full stack reconstruction of the methyl green-counter-stained embryo 16.5 h after the beginning of the time-lapse experiments is shown. Corresponding to Fig. 4; see also Videos S4,6-8.

Video S6. Retinal axons from *slit2*, *slit3* and *slit2/slit3* double crispants present navigation errors around the midline III. Confocal time-lapse (4D) maximum intensity projection images of the optic chiasm region from an *atoh7*:GFP transgenic embryo injected with *slit3* sgRNA/nCas9n, acquired every 15 min from 30 hpf. At the end, a full stack reconstruction of the methyl green-counter-stained embryo 16.5 h after the beginning of the time-lapse experiments is shown. Corresponding to Fig. 4; see also Videos S4,5,7,8.

Video S7. Retinal axons from *slit2*, *slit3* and *slit2/slit3* double crispants present navigation errors around the midline IV. Confocal time-lapse (4D) maximum intensity projection images of the optic chiasm region from an *atoh7*:GFP transgenic embryo

injected with *slit2+slit3* sgRNA/nCas9n, acquired every 15 min from 30 hpf. At the end, a full stack reconstruction of the methyl green-counter-stained embryo 16.5 h after the beginning of the time-lapse experiments is shown. Corresponding to Fig. 4; see also Videos S4-6,8.

Video S8. Retinal axons from *slit2*, *slit3* and *slit2/slit3* double crispants present navigation errors around the midline V. Confocal time-lapse (4D) maximum intensity projection images of the optic chiasm region from an *atoh7*:GFP transgenic embryo injected with *slit2+slit3* sgRNA/nCas9n, acquired every 15 min from 30 hpf. At the end, a full stack reconstruction of the methyl green counter-stained embryo 16.5 h after the beginning of the time-lapse experiments is shown. Corresponding to Fig. 4; see also Videos S4-7.
