# Supplementary figures and images for: Zebrafish Slit2 and Slit3 Act Together to Regulate Retinal Axon Crossing at the Midline
Source: J Dev Biol. 2022 Sep 23;10(4):41. doi: 10.3390/jdb10040041 (PMC9590056; doi:10.3390/jdb10040041)

Figure S2

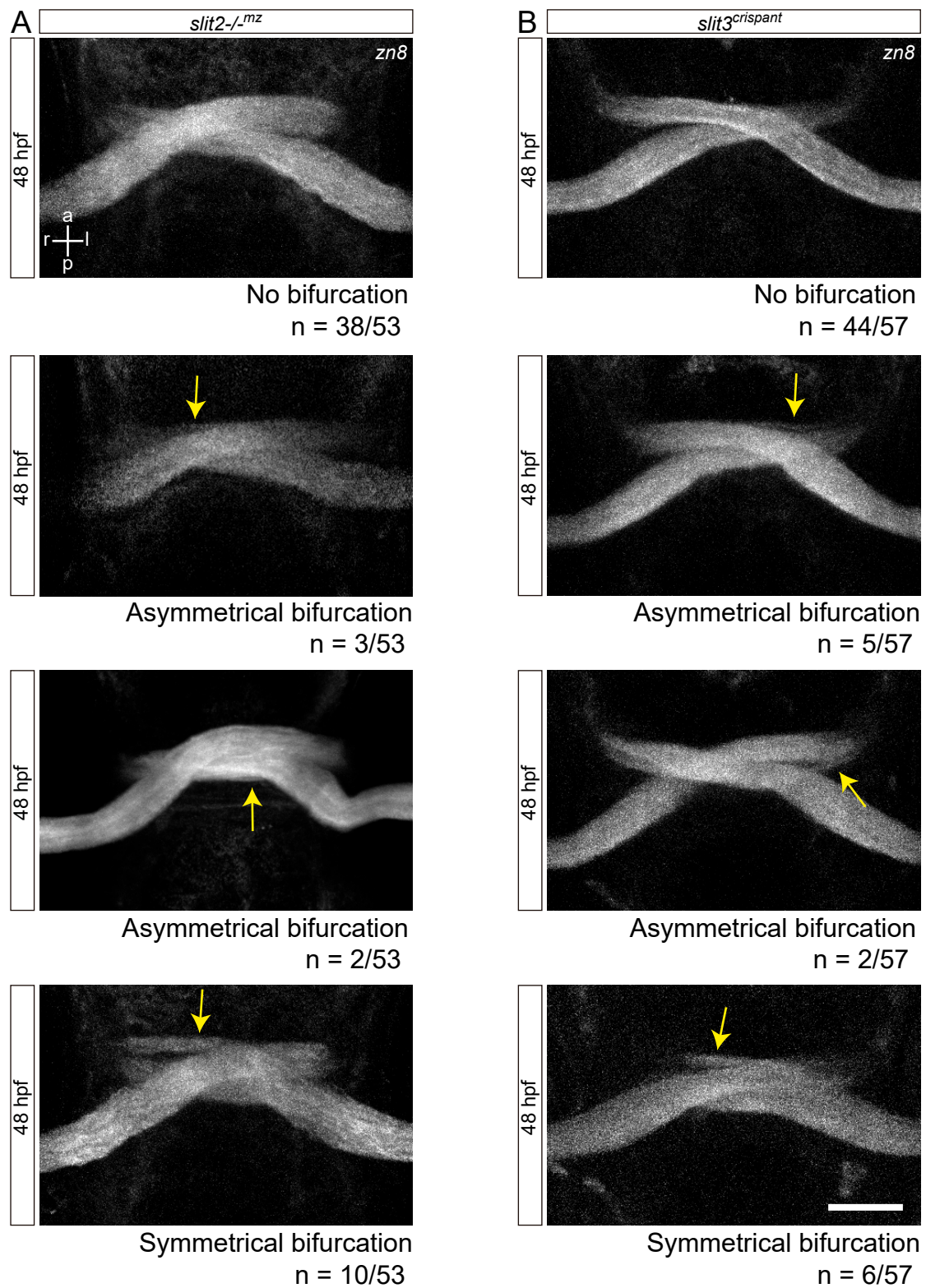

Supplement: Supplementary file 1 [file jdb-10-00041-s001.zip › jdb-1888759-supplementary/Fig. S2rev.pdf]

Figure S3

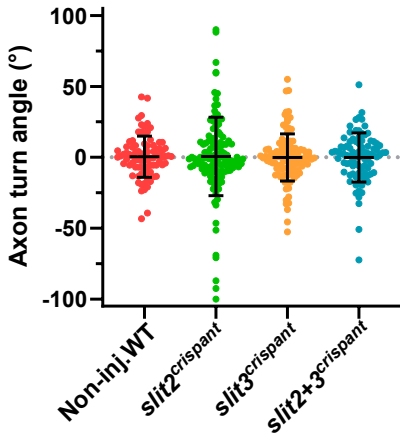

Supplement: Supplementary file 1 [file jdb-10-00041-s001.zip › jdb-1888759-supplementary/Fig. S3rev.pdf]
